# Low diversity of *Angiostrongylus cantonensis* complete mitochondrial DNA sequences from Australia, Hawaii, French Polynesia and the Canary Islands revealed using whole genome next-generation sequencing

**DOI:** 10.1186/s13071-019-3491-y

**Published:** 2019-05-16

**Authors:** Barbora Červená, David Modrý, Barbora Fecková, Kristýna Hrazdilová, Pilar Foronda, Aron Martin Alonso, Rogan Lee, John Walker, Chris N. Niebuhr, Richard Malik, Jan Šlapeta

**Affiliations:** 1 Sydney School of Veterinary Science, University of Sydney, Sydney, NSW 2006 Australia; 2 Department of Pathology and Parasitology, University of Veterinary and Pharmaceutical Sciences Brno, Palackého třída 1946/1, 612 42 Brno, Czech Republic; 3 Institute of Vertebrate Biology, Czech Academy of Sciences, Květná 8, 603 65 Brno, Czech Republic; 4 Institute of Parasitology, Biology Center of the Czech Academy of Sciences, Branišovská 1160/31, 370 05 České Budějovice, Czech Republic; 5 CEITEC VFU, University of Veterinary and Pharmaceutical Sciences Brno, Palackého třída 1946/1, 612 42 Brno, Czech Republic; 6 Instituto Universitario de Enfermedades Tropicales y Salud Pública de Canarias, Universidad de La Laguna, C/Astrofisico F Sanchez, s/n, Tenerife, 38203 La Laguna, Canary Islands Spain; 7 Department Obstetricia y Ginecología, Pediatría, Medicina Preventiva y Salud Pública, Toxicología, Medicina Legal y Forense y Parasitología, Universidad de La Laguna, 38203 San Cristóbal de La Laguna, Canary Islands Spain; 8 Westmead Clinical School, University of Sydney, Sydney, NSW 2145 Australia; 9 Marie Bashir Institute for infectious Diseases and Biosecurity, University of Sydney, Sydney, NSW 2006 Australia; 10 USDA-APHIS-WS, National Wildlife Research Center, Hawaii Field Station, PO Box 10880, Hilo, HI 96721 USA; 11 Centre for Veterinary Education, University of Sydney, Sydney, NSW 2006 Australia; 12 Present Address: Manaaki Whenua-Landcare Research, PO Box 69040, Lincoln, 7608 New Zealand

**Keywords:** Rat lungworm, Mitochondrial genome, Genetic diversity, Invasive species, Next-generation sequencing, Rat lungworm, *cox*1, *Rattus*

## Abstract

**Background:**

Rats (*Rattus* spp.) invaded most of the world as stowaways including some that carried the rat lungworm, *Angiostrongylus cantonensis*, the cause of eosinophilic meningoencephalitis in humans and other warm-blooded animals. A high genetic diversity of *A. cantonensis* based on short mitochondrial DNA regions is reported from Southeast Asia. However, the identity of invasive *A. cantonensis* is known for only a minority of countries. The affordability of next-generation sequencing for characterisation of *A. cantonensis* genomes should enable new insights into rat lung worm invasion and parasite identification in experimental studies.

**Methods:**

Genomic DNA from morphologically verified *A. cantonensis* (two laboratory-maintained strains and two field isolates) was sequenced using low coverage whole genome sequencing. The complete mitochondrial genome was assembled and compared to published *A. cantonensis* and *Angiostrongylus malaysiensis* sequences. To determine if the commonly sequenced partial *cox*1 can unequivocally identify *A. cantonensis* genetic lineages, the diversity of *cox*1 was re-evaluated in the context of the publicly available *cox*1 sequences and the entire mitochondrial genomes. Published experimental studies available in Web of Science were systematically reviewed to reveal published identities of *A. cantonensis* used in experimental studies.

**Results:**

New *A. cantonensis* mitochondrial genomes from Sydney (Australia), Hawaii (USA), Canary Islands (Spain) and Fatu Hiva (French Polynesia), were assembled from next-generation sequencing data. Comparison of *A. cantonensis* mitochondrial genomes from outside of Southeast Asia showed low genetic diversity (0.02–1.03%) within a single lineage of *A. cantonensis*. Both *cox*1 and *cox*2 were considered the preferred markers for *A. cantonensis* haplotype identification. Systematic review revealed that unequivocal *A. cantonensis* identification of strains used in experimental studies is hindered by absence of their genetic and geographical identity.

**Conclusions:**

Low coverage whole genome sequencing provides data enabling standardised identification of *A. cantonensis* laboratory strains and field isolates. The phenotype of invasive *A. cantonensis*, such as the capacity to establish in new territories, has a strong genetic component, as the *A. cantonensis* found outside of the original endemic area are genetically uniform. It is imperative that the genotype of *A. cantonensis* strains maintained in laboratories and used in experimental studies is unequivocally characterised.

**Electronic supplementary material:**

The online version of this article (10.1186/s13071-019-3491-y) contains supplementary material, which is available to authorized users.

## Background

Biological invasions are a recognised outcome of global change [1, 2]. Historically, non-native species were introduced into new areas by the movement of people and goods, [3–6]. The rat (*Rattus* spp.) is recognised globally as an invasive species (GISD 2018: http://www.iucngisd.org) [7, 8]. Rats were accompanied by their pathogens, including viruses, bacteria, protozoans, helminths and ectoparasites [9]. The rat lungworm, *Angiostrongylus cantonensis* (Strongylida: Metastrongylidae), is a cause of eosinophilic meningoencephalitis in humans and animals [10]. The first documented outbreaks of human disease due to *A. cantonensis* occurred in Tahiti and Hawaii in the 1950s [11, 12]. The nematode *A. cantonensis* was described from rat lungs in the Guangzhou region in China and Taiwan more than 30 years earlier [13, 14], with the first human case reported during World War 2 (WW2) in a patient domiciled in Taiwan.

The life-cycle of *A. cantonensis* is sustained between rats, where the adult helminths are confined to the pulmonary arteries and right ventricle, and a range of molluscs, crustaceans, planarians and for example frogs serving as intermediate and transport hosts, respectively [15–17]. Humans, companion animals and wildlife are accidental dead-end hosts for *A. cantonensis*. Although such infections are unimportant to the sustainability of *A. cantonensis*, they may lead to severe, even fatal, infections in these accidental hosts [18–20]. One theory concerning the emergence of *A. cantonensis* outside Southeast Asia following WW2 states that invasion was facilitated by the introduction of the giant African snail, *Achatina fulica* that permitted the parasite to flourish. Alternatively, *A. cantonensis* might have been carried within the snail across the Pacific, where rats had already been established [21–23]. Reports of newly invaded territories keep occurring, e.g. Canary Islands (2010), Uganda (2012) and Oklahoma, USA (2015), although the exact timing and the route of the invasions remains largely uncharacterised [24–26].

Southeast Asia (including China, Taiwan, Vietnam, Thailand, Laos, Cambodia and Myanmar) is considered to be the original endemic region for *A. cantonensis* [21, 22, 27–35]. In Japan, Tokiwa et al. [33] suggested colonization of the area by multiple genetic lineages spreading from the south to the north of Japan. The majority of phylogeographic studies have local character and are based on partial sequences [36]. Data on genetic and phenotypic diversity of *A. cantonensis* within invaded regions remain scarce [10, 37–40]. A recent study has shown that the pathogenicity of *A. cantonensis* for its laboratory host(s) varied between different *A. cantonensis* genetic lineages [41]. The range of *A. cantonensis* phenotypes in rats and molluscs from inside and outside of Southeast Asia may imply that only certain *A. cantonensis* genetic lineages are capable of invading other territories.

**Fig. 1:**
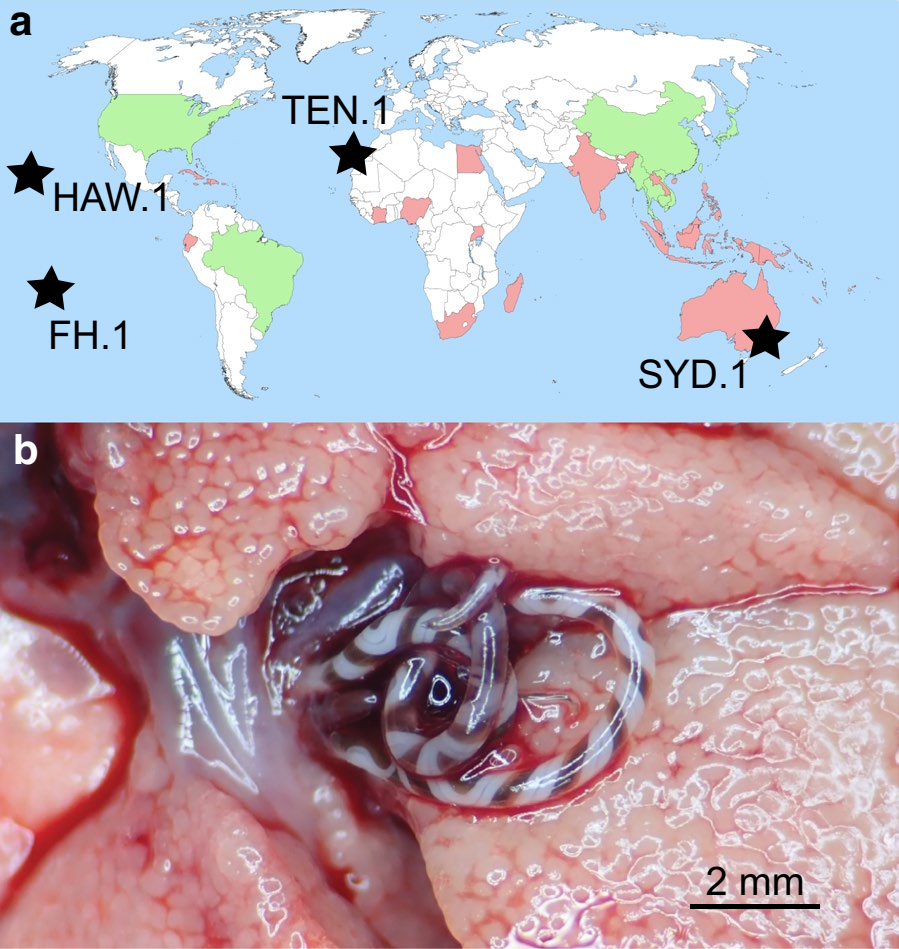
Geographical distribution of *Angiostrongylus cantonensis* (**a**) and localization within a rat (**b**). Locality of material examined in this study (black stars). The original endemic region of *A. cantonensis* is Southeast Asia (China, Taiwan, Vietnam, Thailand, Laos, Cambodia and Myanmar). Countries where *A. cantonensis* is present (**a**) and *cox*1 sequence is publicly available (green). Countries where *A. cantonensis* is present but with no *cox*1 genetic confirmation (red). Several adult females of *A. cantonensis* inhabiting the pulmonary artery (which has been slit open) of Polynesian rat (*Rattus exulans*) from Hawaii Island, Hawaii (**b**). The map was created based on references cited by Barratt et al. [10]

The aim of this study was to determine the extent of mitochondrial DNA (mtDNA) diversity of *A. cantonensis* outside Southeast Asia. We used whole genome low coverage next-generation sequencing to obtain complete mtDNA of *A. cantonensis* originating from four geographically distant regions where it represents an important public health concern: (i) Sydney (Mosman, NSW, Australia); (ii) Fatu Hiva Island (Marquesas, French Polynesia); (iii) Hawaii Island (Hawaii, USA); and (iv) Tenerife (Canary Islands, Spain) (Fig. 1). The low mtDNA diversity of invadin*g A. cantonensis* observed prompted us to review published experimental studies to determine the identity of *A. cantonensis* isolates used in experimental studies. The suitability of partial *cox*1 to unequivocally identify genetic lineages for *A. cantonensis* was evaluated in the context of all available sequence data and the entire mtDNA genome.

## Results

### *Angiostrongylus cantonensis* from Pacific and Atlantic regions determined by morphology and *cox*1 sequence

**Fig. 2:**
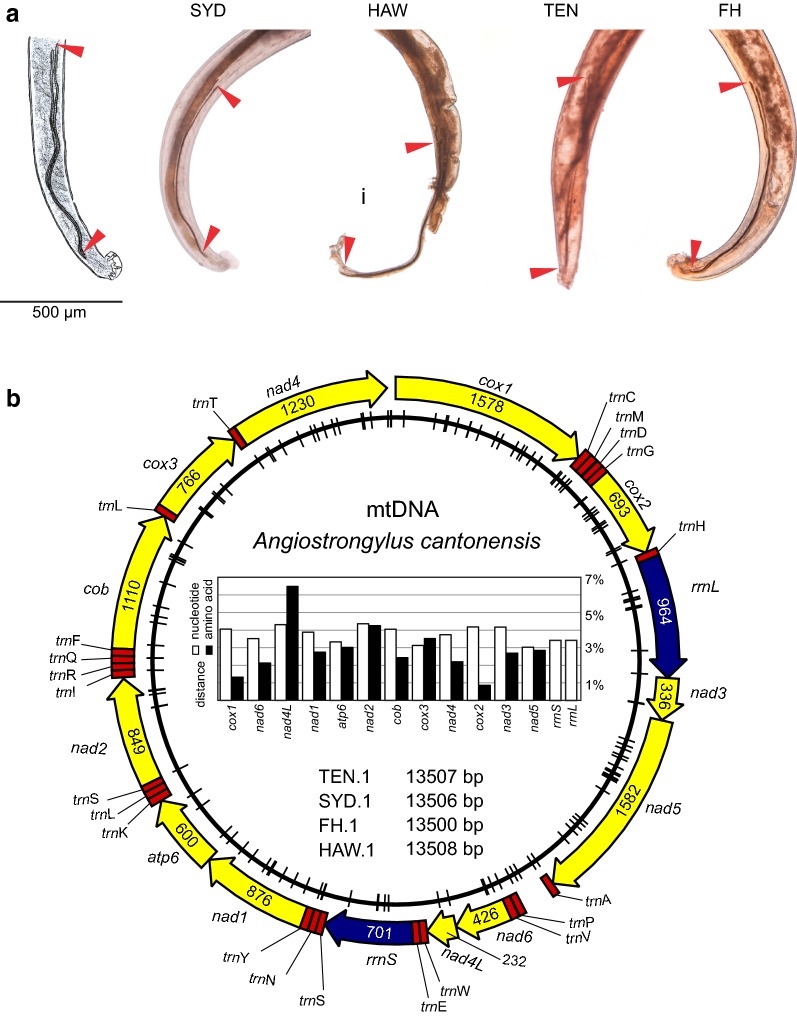
*Angiostrongylus cantonensis* from Sydney, Australia (SYD), Hawaii, USA (HAW), Tenerife, Spain (TEN) and Fatu Hiva, French Polynesia (FH). Caudal parts of male *A. cantonensis* specimens and the original illustration (left) of the species [13]. Length of the spicules (red arrowheads). The tail of the HAW male is damaged, with no effect on the spicules structure (**a**). Diagram of mitochondrial genomes of *A. cantonensis* obtained in our study with defined genes (length indicated on the annotation) and *trn* regions. Dashes on the inner circle localize SNP sites across sequenced vouchers (SYD.1, HAW.1, TEN.1, FH.1). The graph shows the highest pairwise nucleotide and amino acid sequence distances (**b**)

Adults of *A. cantonensis* from the Pacific region (Fatu Hiva, Marquesas, French Polynesia; Hawaii Island, Hawaii, USA; Sydney, Australia) and Atlantic region (Tenerife, Canary Islands, Spain) were morphologically consistent with the original description and re-description of *A. cantonensis sensu* Chen, 1935 and *sensu* Bhaibulaya, 1968, respectively [13, 42]. The spicules of the caudal end of *A. cantonensis* males examined exceeded 1 mm [Sydney specimen: 1.2–1.5 mm (SYD, *n* = 6); Fatu Hiva specimen: 1.1–1.2 mm (FH, *n* = 3); Hawaii specimen: 1.3 mm (HAW, *n* = 1); Canary Islands: 1.2–1.3 mm (TEN, *n* = 5), Fig. 2a]. Partial *cox*1 sequences (primers LCO1490 and HCO2160) of selected vouchers from each series were > 98% identical to the reference *A. cantonensis cox*1 sequence (strain AC3; KT947978).

### Low diversity of four invasive *Angiostrongylus cantonensis* mitochondrial genomes (mtDNA)

Low coverage whole genome next-generation sequencing of *A. cantonensis* DNA (quantity 36–57 ng) yielded 1.4–5.4 G bp with 89.49–92.99% high quality Q30 of the nucleotides (Table 1). Using MitoBIM, we assembled four (SYD.1, HAW.1, FH.1, TEN.1) complete mtDNA sequences, 13,500–13,508 bp (Fig. 2b). Three independent MitoBIM runs, each initially baited with *A. cantonensis* mtDNA (NC_013065), returned identical mtDNA sequences. With the aid of the MITOS Web Server and subsequent manual correction, 12 protein-coding genes representing 3426 amino acids (10,278 bp), 22 *trn* regions and two ribosomal subunits were annotated (Fig. 2b). The partial *cox*1 sequence obtained by PCR (primers LCO1490-HCO2160, *c.*650 bp) from the same initial DNA following Sanger sequencing was 100% identical to the mtDNA assembled from the NGS data.

**Table 1:** Whole genome next-generation sequencing of *Angiostrongylus cantonensis* raw data summary

Identifier	Sequence ID	DNA amount (ng)	Total read bases (G bp)	Total reads (mil)	G-C content (%)	A-T content (%)	Q30 (%)
SYD.1	JS4458	43	2.0	20.2	41.39	58.61	92.28
HAW.1	JS4459	36	5.4	53.5	41.38	58.62	89.49
TEN.1	6967	57	2.3	22.7	41.81	58.19	92.72
FH.1	R23-F	50	1.4	13.8	41.56	58.44	92.99

*Note:* Paired end 101-bp Illumina sequencing of Nextera XT DNA library. Q refers to Phred Quality Score which is calculated with − 10log_10_P, where P is probability of erroneous base call. Q30 stands for 1 incorrect base call in 1000

**Fig. 3:**
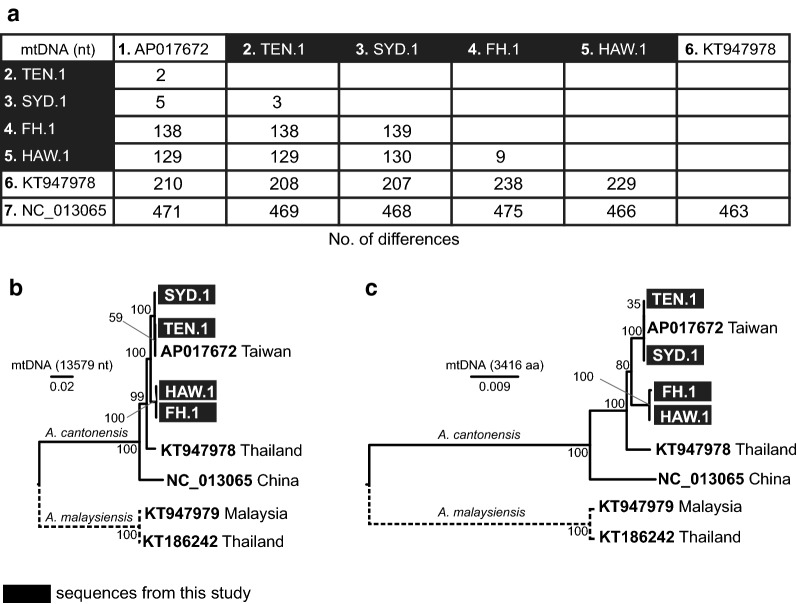
Comparison of seven available complete mtDNA genomes of *Angiostrongylus cantonensis.* Pairwise sequence distance for all available complete mtDNA sequences (13,525 bp) of *A. cantonensis* expressed as number of differences (**a**). Sequence AP017672 originates from Taiwan, KT947978 from Thailand and NC_013065 from China. Maximum likelihood phylogenetic tree reconstructed from complete nucleotide sequences (**b**) by TN93 model [60] and from amino acid sequences (**c**) by JTT model [61]

Nucleotide sequence alignment of four *A. cantonensis* complete mtDNA genomes obtained in this study with three published *A. cantonensis* mtDNA genomes originating from Southeast Asia [Taiwan (AP017672), Thailand (Isolate AC3, KT947978), China (NC_013065)] was reconstructed (length 13,525 nt). Overall, the four new *A. cantonensis* mtDNA sequences (SYD.1, TEN.1, HAW.1, FH.1) differed in ≤ 0.02% (3–139 residues) (Fig. 3a), the SYD.1 and TEN.1 differing in only 3 residues (synonymous mutations C/T and G/T located in *cox*1 and *cox*2, respectively, and a single nucleotide change in *trn*N). Similarly, HAW.1 and FH.1 differed in 9 residues: seven indels in *rrn*L, one indel in a non-coding region between *trn*P and *trn*A, and one mutation C/T in *cob* (position 815) resulting in amino acid A in HAW.1 and V in FH.1, respectively. Compared to published Southeast Asian mtDNA sequences, the greatest similarity was between our isolates and the Taiwanese isolate AP017672, comprising two residue differences (one in *rrn*L and the other in a non-coding region) from TEN.1 to 138 residues for FH.1 (99 located in protein-coding genes). The highest difference was observed between the Chinese isolate NC_013065 and all the other isolates (463–475 residues/3.4–3.5%) (Fig. 3a).

The maximum likelihood phylogenetic trees reconstructed from complete mtDNA nucleotide (13,579 nt; Fig. 3b) and amino acid sequences (3,416 aa; Fig. 3c) showed clear separation of *A. cantonensis* and *A. malaysiensis* clades. The *A. cantonensis* clade was further divided in four sub-clades comprising (i) Chinese isolate (NC_013065); (ii) Thailand isolate (KT947978); (iii) isolates HAW.1, FH.1; and (iv) including Taiwanese isolate (AP017672), SYD.1 and TEN.1 isolates. All four sub-clades were supported by high bootstrap values (99–100 for nucleotide and 80–100 for amino acid tree; Fig. 3b, c).

The nucleotide pairwise sequence distance percentages (PSD_N_) calculated for each of the protein-coding genes and rRNA gene subunits varied from 3.0% (*nad*5) to 4.4% (*nad*2) (Fig. 2b). The highest amino acid pairwise sequence distance percentage (PSD_AA_) was observed in the shortest gene *nad*4L (77 aa). Interestingly, six of seven analysed sequences were identical in this gene, only the Chinese isolate (NC_013065) differed from the others in 5 amino acids. The lowest PSD_AA_ was observed in *cox*2 (maximum difference 0.9%), representing two polymorphic sites (out of 230) where serine substituted for either asparagine or proline. The second lowest PSD_AA_ was detected in *cox*1 (maximum difference 1.3%). Six of seven *cox*1 sequences (525 aa), differed by a single amino acid (V/I in position 516). The sequence of the Chinese isolate (NC_013065) differed from the other six sequences in 6–7 amino acids. The interspecific PSD_AA_ of *cox*1 sequences between *A. cantonensis* and *A. malaysiensis* (KT947979) was 12–14 residues (2.3–2.7%).

### Short overlapping *cox*1 sequence limits characterization of rat lungworm *Angiostrongylus cantonensis*

**Fig. 4:**
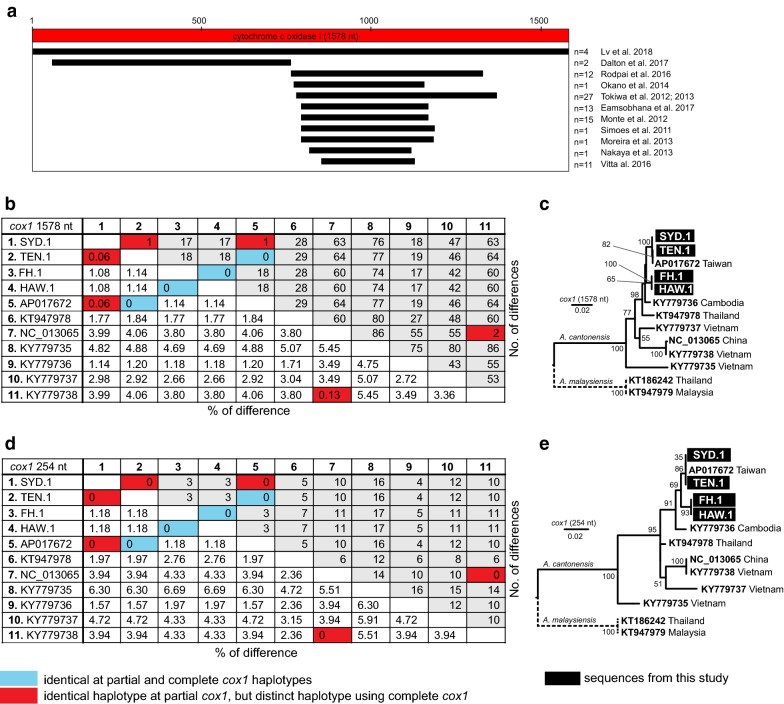
Comparison of *Angiostrongylus cantonensis* diversity at *cox*1 sequences. Map of *cox*1 regions amplified by different authors relative to the complete *cox*1 (**a**). Pairwise sequence distance expressed as percentage of difference and number of differences for complete *cox*1 (**b**). The alignment of complete *cox*1 included 1,433 (90.1%) conserved, 145 (9.2%) variable and 75 (4.8%) parsimony informative sites and 70 (4.4%) singletons. Maximum likelihood tree reconstructed using the TN93 model [60] from this alignment (**c**). Pairwise sequence distance expressed as percentage of difference and number of differences for 254-bp region of *cox*1, where the majority of available sequences overlap (**d**). Alignment of the 254-bp region included 227 (89.4%) conserved, 27 (10.6%) variable and 13 (5.1%) parsimony informative sites and 14 (5.5%) singletons. Maximum likelihood tree reconstructed using the TN93 model [60] from this alignment (**e**). In both trees, bootstrap values above 50 are shown

Currently, there are 86 *A. cantonensis* (including 3 complete mtDNA) and 13 *A. malaysiensis* (including 2 complete mtDNA) *cox*1 sequences available in public DNA repositories. Six sequences of *A. cantonensis* (GU138106–11) were excluded from further analyses because they included internal stop codons. The complete coding sequences of *cox*1 (1578 bp; 525 aa + stop codon) extracted from newly obtained complete mtDNA were aligned with available sequences, demonstrating no gaps and no variation in length (Additional files 1, 2, 3, 4, 5). There were 13 complete *cox*1 sequences (11 for *A. cantonensis*, 2 for *A. malaysiensis*) in the final alignment (Fig. 4a, b, c). The remaining sequences were distributed across ten different fragments of *cox*1 depending on the primer sets used in the respective studies (Fig. 4a). The majority (98%, 95/97) of the *A. cantonensis cox*1 sequences overlapped in 254-bp region (positions 847–1101). The *cox*1 sequences MF000735-MF000736 (*A. cantonensis* from mainland USA) were outside this 254-bp region (Fig. 4a).

The comparison between PSD_N_ for 11 available complete *cox*1 and restricted to the 254-bp fragment of the same sequences demonstrated a decrease in the absolute number of polymorphic nucleotide residues (Wilcoxon matched pair test, *P*-value < 0.0001) (Fig. 4b, d). While sequences SYD.1, TEN.1 and AP017672 represented different haplotypes of complete *cox*1, in this 254-bp region, they appeared identical. Similarly, sequences KY779738 and NC_013065 were not detected as different haplotypes based on this 254-bp region. The phylogenetic tree reconstructed from the 254-bp alignment of *cox*1 (Fig. 4e) showed lower resolution of internal relationships within the *A. cantonensis* clade compared to the tree reconstructed from complete *cox*1 (Fig. 4c).

**Fig. 5:**
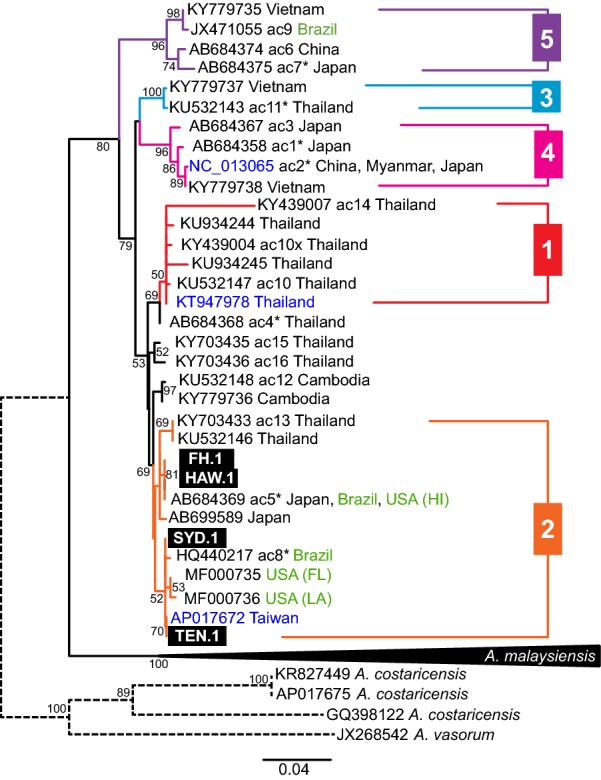
Phylogenetic analysis of *Angiostrongylus cantonensis* at *cox*1. A maximum likelihood tree was inferred using maximum likelihood (GTR+G [59]; bootstrap 100). Each sequence represents a unique haplotype. Bootstrap values noted at the nodes, only values above 50 are shown. Previously named haplotypes (ac1-16) marked. Haplotypes comprising multiple sequences are marked by asterisks. Sequences in green originate outside of the original endemic region of *A. cantonensis*. Previously published complete mtDNA sequences are presented in blue. Numbering of the subclades is based on Dusitsittipon et al. [36] The clade of *A. malaysiensis* is collapsed as the species was not focus of our study. The full tree is provided in Additional file 7

To show relationships between our four newly sequenced isolates and previously published data, the initial dataset was narrowed down to 56 unique *cox*1 sequences (*A. cantonensis*, *n* = 40; *A. malaysiensis*, *n* = 12; outgroup, *n* = 4) and used to construct a maximum likelihood phylogenetic tree. The alignment consisted of 1581 characters with 1094 (69.1%) conserved, 484 (30.6%) variable and 316 (20%) parsimony informative sites and 168 (10.6%) singletons. Tree topology (Fig. 5) showed separation of *A. malaysiensis* and *A. cantonensis* as two distinct clades. Eight sequences named as *A. cantonensis* downloaded from GenBank clustered within the *A. malaysiensis* clade (Additional files 6 and 7). These sequences originated from Thailand (*n* = 7) and Taiwan (*n* = 1).

All of our four *A. cantonensis* isolates represented by *cox*1 clustered together with sequence AP017672 (extracted from mtDNA) to the Clade 2 *sensu* Dusitsittipon et al. [36]. This clade also contained sequences originating from Brazil, Hawaii and continental USA (Florida and Louisiana). Further subdivision of the subclade was not supported (bootstrap < 50%).

### Systematic literature review shows lack of complete characterization of experimentally studied *Angiostrongylus cantonensis* strains

The Sydney and Fatu Hiva *A. cantonensis* strains are currently maintained in experimentally infected laboratory rats and snails (Table 2). The literature search yielded 412 articles on *A. cantonensis* published after 2011, with 104 articles describing experimental work (Additional file 8). The exact origin of *A. cantonensis* used for the experimental studies was specified in 46% of studies (48/104) and 4% (2/48) provided *cox*1 to confirm the identity of the parasite (Table 3).

**Table 2:** Summary of *Angiostrongylus cantonensis* used in this study

Identifier	Locality of origin, host	Date of collection for analysis	Laboratory strain (laboratory host, year of isolation)
FH.1	Fatu Hiva, Marquesas Islands, French Polynesia, *Rattus exulans*	February 2017	University of Veterinary and Pharmaceutical Sciences, Brno, Czech Republic, Wistar rat, *Rattus norvegicus*, 2017
TEN.1	Tenerife, Canary Islands, Spain; *Rattus norvegicus*	April 2018	–
HAW.1	Hawaii Island, Hawaii, USA; *Rattus exulans*	May, 2018	–
SYD.1	Mosman (near Taronga Zoo, Sydney), NSW, Australia; *Rattus norvegicus*	December, 2017	Westmead Hospital, NSW, Australia; Wistar rat, *Rattus norvegicus*, 1997

**Table 3:** Review of identification of *A. cantonensis* strains used in experimental studies

	Total	Vertebrate host				Invertebrate host	
		Diagnostics	Pathology	Physiology	Treatment	Pathology	Physiology
Number of articles	104	3	46	31	12	11	1
Origin of the strain specified	48	1	14	17	5	10	1
Helminths sequenced	2	0	1	1	0	0	0
*cox*1 haplotype determined	1	0	0	1	0	0	0

## Discussion

To demonstrate how whole genome sequencing can assist routine nematode characterization, we analysed complete mtDNA genomes of the invasive metastrongylid *A. cantonensis* originating from four geographically distant areas well outside the endemic range of the parasite in Southeast Asia. Dusitsittipon et al. [36] attempted to synthesize published data on *A. cantonensis* mtDNA and concluded that detailed morphological and genetic characterization is urgently needed. Our data not only provide complete mtDNA of morphologically identified *A. cantonensis* vouchers, but the sequence data represent a resource from which other target genes sequences can be extracted.

The circular mtDNA of our *A. cantonensis* includes two ribosomal subunits, 12 protein-coding genes and 22 *trn* coding regions, concordant with published results [43, 44]. The generally low mtDNA genetic distance amongst invasive *A. cantonensis* isolates studied is in stark contrast to the genetic distances observed between Southeast Asian isolates, suggesting that only a limited number of mtDNA haplotypes had the capacity to emerge as globally invasive *A. cantonensis* [36, 41].

The isolates of *A. cantonensis* from tropical Pacific islands of Fatu Hiva and Hawaii are almost identical. Similarly, two localities which are almost 20,000 km apart (Sydney and Tenerife) differ by only 3 nucleotides across the entire 13.5 kbp mtDNA. *Angiostrongylus cantonensis* was first detected in eastern Australia in 1950s, while Tenerife, Canary Islands has only recently been invaded [24, 45, 46]. It would be too speculative to suggest an Australian origin for the recent Tenerife invasion, because we do not know the extent of *A. cantonensis* diversity in other regions that could have equally been the source population, and there is no plausible historical connection between Australia and the Canary Islands (Fig. 1). Indeed, the Tenerife and Sydney isolates are almost identical to the Taiwanese isolate (AP017672) from BioProject PRJEB493. We speculate that the rapid spread of *A. cantonensis* in the Pacific region was a consequence of naval operations during and/or after the World War II, because troop and supply ships could readily permit spread of the *A. cantonensis* infected rats and/or snails. In a telling analogy, *A. cantonensis* repeatedly colonised Japan during the 20th century, spreading from southern islands to the north resulting in a presence of several different haplotypes from three clades [22].

The phylogeny inferred from partial *cox*1 sequences corresponds to the tree topography described by Dusitsittipon and colleagues [36]. Numerous haplotypes from different clades detected in China, Taiwan, Thailand, Vietnam, Myanmar and Cambodia support the theory that *A. cantonensis* originated historically from Southeast Asia in the same manner as its *Rattus* hosts [10, 32–34, 47]. In contrast, all but one sequence originating from outside of the endemic range of *A. cantonensis* cluster within the Clade 2 *sensu* Dusitsittipon et al. [36], confirming the trend of low diversity for invasive *A. cantonensis* isolates. The situation in Hawaii might have experienced more complicated scenario, because *cob* sequences KP721454–55 are distinct from our HAW.1 and exhibit high similarity to the Chinese isolate NC_013065 (Additional file 9). Unfortunately, no detailed information is available on the origin of the previously submitted *A. cantonensis* (e.g. the particular island) from Hawaii.

The genotypic diversification of a parasite is commonly associated with morphological and biological diversification, enabling the organism to acquire traits that permit invasion of new territories and hosts [48, 49]. The complexity of haplotypes of *A. cantonensis* suggests variability in its biological traits, but this is yet to be established experimentally [36]. The review of 104 experimental studies involving *A. cantonensis* revealed that only two studies identified their *A. cantonensis* genetically using, for example, *cox*1 [41, 50]. Nevertheless, Lee et al. [50] while comparing infectivity and pathogenicity between what they considered two *A. cantonensis* (named strain P and H) inadvertently worked with two distinct *Angiostrongylus* species, because *cox*1 (strain H; KF591126) clusters within the *A. malaysiensis* clade in our analyses (Additional file 7).

In the past, obtaining the complete mtDNA of *A. cantonensis* was accomplished with laborious PCR and primer walking [43, 51]. With the accessibility of next-generation sequencing services, it is logical to consider this methodology for any *A. cantonensis* laboratory-maintained strain. Such resource can be further mined for other genes of interest, including those under Darwinian selection and therefore informing on phenotypic characterization.

## Conclusions

Using whole genome next-generation sequencing methods, we assembled and analysed complete mtDNA of four *A. cantonensis* isolates originating from four geographically disparate localities outside of the parasite’s endemic range. The observed uniformity of invasive strains implies that only certain *A. cantonensis* genetic lineages have the capacity to become globally invasive. Our review of published experimental studies demonstrated a need of improved consistency in reporting the identity and origin of tested *A. cantonensis*. We encourage researchers working with *A. cantonensis* in laboratories to provide thorough information on their strains, including the origin (country, region, host species) and genetic characterization represented by sharing raw data, or mtDNA sequence or minimally, provide a sequence of complete *cox*1. Having a resource with standardised good quality information will provide a basis for future studies focused on phenotypic traits of *A. cantonensis*.

## Methods

### Collection of adult rat lungworms *Angiostrongylus cantonensis*

The adults of *A. cantonensis* were collected from *Rattus* spp. in four geographically distant localities in the Pacific and Atlantic region and donated for the study as vouchers in 90% ethanol (Fig. 1a, Table 2).

### Morphological determination of adult rat lungworms *Angiostrongylus cantonensis*

All the adult specimens were examined under the light microscope (Olympus BX53 and BX60) equipped with Nomarski interference contrast optics. The morphology was compared to descriptions of *A. cantonensis*, *A. mackerrasae* and *A. malaysiensis* [13, 42, 52, 53]. In male worms, the structure of the copulatory bursa was observed, and spicules were measured as the spicules varying in length from 1.0–1.46 mm, postero-lateral ray significantly shorter than medio-lateral ray and separation of the ventro-ventral from the latero-ventral ray at the point about distal one third of the common trunk are typical features for distinguishing *A. cantonensis*. Females of *A. cantonensis* are determined by the length of the vagina in the range 1.5–3.25  mm (2.1 mm on average), by the vulva located 0.16 mm from the anus and finally, by the absence of a minute terminal projection at the tip of the tail [42, 52].

Several specimens from each series were deposited to the helminthological collection of the Institute of Parasitology, Biology Center, České Budějovice (Fatu Hiva and Tenerife material; accession number IPCAS N-260) and to the Australian National Wildlife Collection, CSIRO, Canberra, Australian Capital Territory, Australia (Hawaii and Sydney material; accession numbers: W/L HC# N5703–N5711)

### Isolation of genomic DNA from tissue segment of adult rat lungworms *Angiostrongylus cantonensis*

DNA was isolated from dried *c.*0.5 cm segment of the mid-body using the Nucleospin Tissue XS (Macherey-Nagel, Düren, Germany) or Isolate II Genomic DNA Kit (Bioline, Alexandria, Australia) according to manufacturers’ instructions and eluted in 100 µl of Tris buffer (pH = 8.5). DNA was stored at − 20 °C.

### Amplification of *cox*1 mtDNA from rat lungworms *Angiostrongylus cantonensis*

Partial sequence of the cytochrome *c* oxidase subunit 1 gene (*cox*1) was amplified using primers LCO1490 (forward) and HCO2198 (reverse) [54]. PCR mixtures were prepared using 15 µl of MyTaqTM Red Mix Kit (Bioline, Alexandria, Australia), 10.5 µl of PCR water, 1.25 µl of each of the primers (10 µM) and 2 µl of the template DNA. The PCR protocol was as follows: initial denaturation at 95 °C for 1 min; 35 cycles of 15 s at 95 °C, 15 s at 55 °C and 10 s at 72 °C; and a final elongation at 72 °C for 7 min. The PCR products were sequenced in Macrogen Inc. (Amsterdam, Netherlands and Seoul, South Korea), the quality of sequences was assessed in CLC Genomic Workbench 6.9.1 (https://www.qiagenbioinformatics.com/) and the sequences were searched through BLAST [55] (https://blast.ncbi.nlm.nih.gov/Blast.cgi) to confirm the identity of the worms.

### Sequencing and assembly of the rat lungworms *Angiostrongylus cantonensis* mtDNA from the whole genome sequencing data

Isolated DNA (36–57 ng; Table 1) was used for Illumina Nextera XT library construction followed by the next-generation sequencing using 100 bp paired end Illumina HiSeq 2500 sequencing systems utilizing at the depth of 1Gb of raw sequence data (Macrogen, Seoul, Republic of Korea). All four specimens used for the whole genome sequencing were females. The complete mitochondrial genome (mtDNA) was assembled from FastQ data using the MITObim pipeline [56] available at https://github.com/chrishah/MITObim with the sequence of *A. cantonensis* complete mtDNA (NC_013065) as bait. The assembly was repeated three times with varying percentage of the raw FastQ sequence data used (2–10%), keeping mtDNA coverage at 60–100×. The obtained mtDNA was annotated with the aid of MITOS Web Server [57] available at http://mitos.bioinf.uni-leipzig.de/ and aligned with available *Angiostrongylus* spp. complete mtDNA sequences in CLC Genomics Workbench 6.9.1. for manual validation.

### Pairwise sequence distance of complete mtDNA sequences

Newly obtained mtDNA sequences were aligned with published mtDNA sequences of *A. cantonensis* (AP017672, KT947978, NC_013065) and pairwise sequence distance (PSD) expressed as number of differences was calculated in CLC Genomics Workbench 6.9.1. Another mtDNA sequence (KT186242) labelled as *A. cantonensis* was not included in our analyses as Dusitsittipon et al. [36] showed that the sequence clusters within the *A. malaysiensis* clade. To assess intraspecific diversity of individual genes and thus their suitability for phylogenetic studies, all 12 mitochondrial genes were separately translated to amino acids and PSD was calculated for both nucleotide (PSD_N_) and amino acid (PSD_AA_) sequences. The PSD_N_ was calculated for both mitochondrial rRNA gene subunits. As *cox*1 is the most commonly used gene for studying diversity and phylogeography of *Angiostrongylus cantonensis*, we included *cox*1 sequence extracted from complete mtDNA of *A. malaysiensis* (KT947979) for PSD_AA_ calculation, aiming to compare the intra- and interspecific variability. The maximum likelihood trees were constructed from complete nucleotide mtDNA sequences and from amino acid sequences of the 12 protein-coding genes in MEGA X [58] using sequences of *A. malaysiensis* (KT186242, KT947979) as outgroup. The model was selected by model test implanted in MEGA X.

### Analysis of *cox*1 alignment and maximum likelihood phylogenetic tree of *cox*1 sequences

Complete sequences of c*ox*1 were extracted from available *A. cantonensis* mtDNA (*n* = 7) and all *cox*1 sequences labelled as *A. cantonensis* were downloaded from GenBank. The geographical location of all isolates was recorded. All sequences were aligned in CLC Genomics Workbench 6.9.1 and the alignment was manually checked for gaps and stop-codons. As we determined that the majority of sequences overlapped in a short fragment (254 nt) of *cox*1, we decided to compare the information contained in this fragment with the information from complete *cox*1. The PSD_N_ expressed as number of differences and percentage of difference was calculated for 11 available complete *cox*1 sequences (7 extracted from complete mtDNA and 4 sequences KY779735–KY779738) and for the overlapping 254-bp fragment of the same 11 sequences. Paired values were compared using Wilcoxon matched pair test (GraphPad Prism 7.02, GraphPad, CA) at significance level *P*-value 0.05. Number of singletons, conserved, variable and parsimony informative sites were recorded, and a maximum likelihood tree was constructed in MEGA X [58] from both alignments using *cox*1 sequences of *A. malaysiensis* (KT186242, KT947979) as the outgroup. The GTR+G model was chosen by model test integrated in MEGA X [58] software. The phylogenetic relationships of the taxa were tested by 100 replicates of bootstrap. To provide corroborating evidence using an alternative gene, we analysed *cob* of *A. cantonensis* (Additional file 9).

To compare diversity of all available *A. cantonensis cox*1 sequences, a maximum likelihood tree was constructed. The alignment comprised one sequence representing each recognised *A. cantonensis* haplotype. As there are apparently sequences (including a mitochondrial genome accession number KT186242), labelled as *A. cantonensis,* which are in fact *A. malaysiensis* (see Dusitsittipon et al. [58]), sequences of *A. malaysiensis* (*n* = 13) were added to the analysis as the *A. cantonensis* sibling species and for the verification of the GenBank sequences taxonomic identity. Complete *cox*1 sequences extracted from mtDNA of *A. costaricensis* (AP017675, GQ398122, KR827449) and *A. vasorum* (JX268542) were used as outgroups. A maximum likelihood tree was constructed in MEGA X [58] by the GTR+G model [59] chosen by the model test implemented within the software. The phylogenetic relationships of the taxa were tested using 100 replicates of bootstrap.

### Systematic review of *A. cantonensis* literature published between 2011–2019

On January 9, 2019, we searched the Web of Science database using the keywords “Angiostrongylus” AND “cantonensis”. Because the first *cox*1 sequence of *A. cantonensis* was published in 2011 [40], we limited the search to articles published between 2011 and 2019. The next step was identification of experimental studies using *A. cantonensis*. The inclusion criteria were: (i) the authors infected any vertebrate or invertebrate host in the laboratory irrespective of which aspect of the infection was being studied, including investigations concerning biological traits of the parasite or even just aspects of the life-cycle of the parasite; and (ii) experiments where *in vitro* cultivation of any parasite stage was the objective of the study. Phylogeographic studies, case reports, reviews or reports of new occurrence were not evaluated as experimental. The decision as to whether the study was or was not experimental was based on reading the abstracts, or the full-text (if it was not clear from the abstract).

Experimental studies were further classified based on the type of the host investigated (vertebrate or invertebrate) and objectives of the study (diagnostics, pathology, physiology, treatment) based on reading the abstract or full-text. As our aim was to determine the amount of information provided on laboratory-maintained strains of *A. cantonensis*, full-texts were manually searched for any information on how the authors obtained the helminths for the experiment(s), including reference to a previous publication, whether the isolate comes from nature/other laboratory or if the isolate is maintained in the laboratory on a long-term basis, etc., and also, if any DNA characterization of the strain was attempted. Thus, the results were tabulated in categories: (i) origin of the helminths specified; (ii) any DNA marker sequenced; (iii) *cox*1 haplotype determined.

## Additional files

**Additional file 1: Alignment S1.** Alignment of complete mtDNA sequences of all available *A. cantonensis* and *A. malaysiensis* in FASTA format.

**Additional file 2: Alignment S2.** Alignment of complete mtDNA sequences of all available *A. cantonensis* and *A. malaysiensis* in CLC format including annotations.

**Additional file 3: Table S1.** Overview of *Angiostrongylus cantonensis cox*1 sequences from GenBank. Sequences in bold represent the given haplotype in the *cox*1 phylogenetic tree (Fig. 5). *Abbreviation*: mtDNA, sequence was extracted from complete mtDNA.

**Additional file 4: Alignment S3.** Alignment of complete *cox*1 sequences used for PSD table and maximum likelihood tree in Fig. 4b, c.

**Additional file 5: Alignment S4.** Alignment of 254-bp fragment of *cox*1 sequences used for PSD table and maximum likelihood tree in Fig. 4d, e.

**Additional file 6: Alignment S5.** Alignment of *cox*1 sequences used for construction of maximum likelihood tree in Fig. 5.

**Additional file 7: Tree S1.** Full maximum likelihood tree of *cox*1 from Fig. 5 where the *A. malaysiensis* clade is not collapsed.

**Additional file 8: Table S2.** List of publications used in our literature review.

**Additional file 9: Text S1.** Analysis of diversity of *cob* in invasive lineages of *A. cantonensis*.

## Abbreviations

aa: amino acid
bp: base pairs
*cox*1: gene for subunit 1 of cytochrome *c* oxidase
*cox*2: gene for subunit 2 of cytochrome *c* oxidase
*cob*: gene for cytochrome *b*
mtDNA: mitochondrial DNA
*nad*2: gene for subunit 2 of NADH dehydrogenase
*nad*5: gene for subunit 5 of NADH dehydrogenase
*nad*4L: gene for subunit 4L of NADH dehydrogenase
NGS: next-generation sequencing
nt: nucleotide
PSD: pairwise sequence distance
PSD_N_: pairwise sequence distance of nucleotide sequences
PSD_AA_: pairwise sequence distance of amino acid sequences
SNP: single nucleotide polymorphism
