# Supplementary figures and images for: Low diversity of Angiostrongylus cantonensis complete mitochondrial DNA sequences from Australia, Hawaii, French Polynesia and the Canary Islands revealed using whole genome next-generation sequencing
Source: Parasit Vectors. 2019 May 16;12:241. doi: 10.1186/s13071-019-3491-y (PMC6524341; doi:10.1186/s13071-019-3491-y)

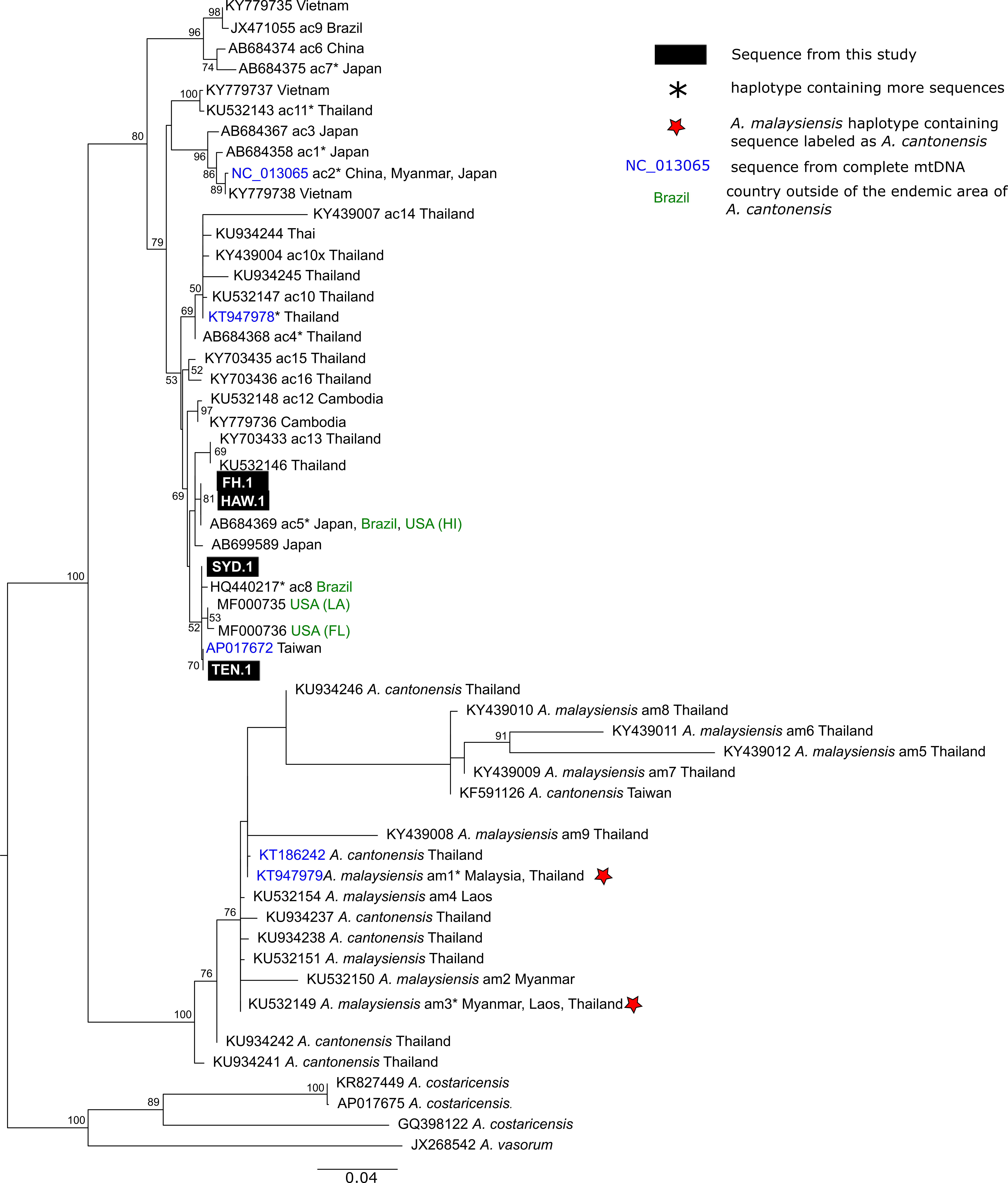

Supplement: Supplementary file 7 — Additional file 7: Tree S1. Full maximum likelihood tree of cox1 from Fig. 5 where the A. malaysiensis clade is not collapsed. [file 13071_2019_3491_MOESM7_ESM.png]
