# Supplementary material for: Low diversity of Angiostrongylus cantonensis complete mitochondrial DNA sequences from Australia, Hawaii, French Polynesia and the Canary Islands revealed using whole genome next-generation sequencing
Source: Parasit Vectors. 2019 May 16;12:241. doi: 10.1186/s13071-019-3491-y (PMC6524341; doi:10.1186/s13071-019-3491-y)
Supplement: Supplementary file 9 — Additional file 9: Text S1. Analysis of diversity of cob in invasive lineages of A. cantonensis. [file 13071_2019_3491_MOESM9_ESM.docx]

**Analysis of *cob* sequences of invasive *A. cantonensis* isolates**

*Pairwise sequence distance of cob sequences*

All *cob* sequences labeled as *A. cantonensis* were downloaded from GenBank and geographical origin was recorded. Downloaded sequences were aligned with *cob* sequences extracted from available mtDNA (n=7) and checked for stop codons. Pairwise sequence distance was calculated for sequences that did not originate in endemic area of the parasite (South-East Asia, Japan).

*Invasive isolates of rat lungworm Angiostrongylus cantonensis show low diversity of cob*

Comparison of PSD of the complete *cob* sequences extracted from available mtDNA showed that the isolates TEN.1 and SYD.1 were identical with sequence AP017672 originating from Taiwan. The isolate HAW.1 differed from FH.1 in a single nucleotide (Table A). The difference between isolates TEN.1/SYD.1 and HAW.1/FH.1 was 10 and 11 bases respectively.

In total, 167 sequences of *cob* labeled as *A. cantonensis* varying in length from 852 to 1110 bp were available in GenBank. The only two sequences that did not originate in the endemic area of *A. cantonensis* were two sequences from Hawaii (accession numbers KP721454–5). The two sequences were identical and when compared to our sequence HAW.1, 32 out of 866 bp were different (Table B). Isolates SYD.1 and TEN.1 differed in 28 bases. The GenBank Hawaiian isolate was most similar to sequences NC_013065 originating from China, the observed difference was 6 out of 866 bp.

**Table SA.** Pairwise sequence distance (PSD) of partial *cob* (1110 bp) extracted from all available complete mtDNA sequences of *A. cantonensis* expressed as a number of different bases.

| 1110 nt | 1. AP017672 | 2. TEN.1 | 3. SYD.1 | 4. FH.1 | 5. HAW.1 | 6. KT947978 |
| --- | --- | --- | --- | --- | --- | --- |
| 2. TEN.1 | 0 |  |  |  |  |  |
| 3. SYD.1 | 0 | 0 |  |  |  |  |
| 4. FH.1 | 11 | 11 | 11 |  |  |  |
| 5. HAW.1 | 10 | 10 | 10 | 1 |  |  |
| 6. KT947978 | 15 | 15 | 15 | 20 | 19 |  |
| 7. NC_013065 | 40 | 40 | 40 | 45 | 44 | 40 |

**Table SB.** Pairwise sequence distance (PSD) of complete *cob* (852 bp) extracted from all available complete mtDNA sequences of *A. cantonensis* and the only other invasive isolate (originating from Hawaii) available in GenBank expressed as a number of different bases.

| 866 nt | 1. AP017672 | 2. TEN.1 | 3. SYD.1 | 4. FH.1 | 5. HAW.1 | 6. KT947978 | 7. NC_013065 |
| --- | --- | --- | --- | --- | --- | --- | --- |
| 2. TEN.1 | 0 |  |  |  |  |  |  |
| 3. SYD.1 | 0 | 0 |  |  |  |  |  |
| 4. FH.1 | 9 | 9 | 9 |  |  |  |  |
| 5. HAW.1 | 8 | 8 | 8 | 1 |  |  |  |
| 6. KT947978 Thailand | 11 | 11 | 11 | 16 | 15 |  |  |
| 7. NC_013065 China | 32 | 32 | 32 | 37 | 36 | 32 |  |
| 8. KP721454 Hawaii | 28 | 28 | 28 | 33 | 32 | 28 | 6 |
